# Comparison of different feature extraction methods for applicable automated ICD coding

**DOI:** 10.1186/s12911-022-01753-5

**Published:** 2022-01-12

**Authors:** Zhao Shuai, Diao Xiaolin, Yuan Jing, Huo Yanni, Cui Meng, Wang Yuxin, Zhao Wei

**Affiliations:** 1 Department of Information Center, Fuwai Hospital, Chinese Academy of Medical Sciences and Peking Union Medical College, Beijing, China; 2 Department of Information Center, Fuwai Hospital, National Center for Cardiovascular Diseases, Chinese Academy of Medical Sciences and Peking Union Medical College, 167 Beilishi Road, Beijing, 100037 China

**Keywords:** Automated ICD coding, Feature extraction, Bag-of-words, BERT, Word2vec, Interpretability

## Abstract

**Background:**

Automated ICD coding on medical texts via machine learning has been a hot topic. Related studies from medical field heavily relies on conventional bag-of-words (BoW) as the feature extraction method, and do not commonly use more complicated methods, such as word2vec (*W2V*) and large pretrained models like *BERT*. This study aimed at uncovering the most effective feature extraction methods for coding models by comparing *BoW*, *W2V* and *BERT* variants.

**Methods:**

We experimented with a Chinese dataset from Fuwai Hospital, which contains 6947 records and 1532 unique ICD codes, and a public Spanish dataset, which contains 1000 records and 2557 unique ICD codes. We designed coding tasks with different code frequency thresholds (denoted as $f_s$), with a lower threshold indicating a more complex task. Using traditional classifiers, we compared *BoW*, *W2V* and *BERT* variants on accomplishing these coding tasks.

**Results:**

When $f_s$ was equal to or greater than 140 for Fuwai dataset, and 60 for the Spanish dataset, the *BERT* variants with the whole network fine-tuned was the best method, leading to a *Micro-F*1 of 93.9% for Fuwai data when $f_s=200$, and a *Micro-F*1 of 85.41% for the Spanish dataset when $f_s=180$. When $f_s$ fell below 140 for Fuwai dataset, and 60 for the Spanish dataset, *BoW* turned out to be the best, leading to a *Micro-F*1 of 83% for Fuwai dataset when $f_s=20$, and a *Micro-F*1 of 39.1% for the Spanish dataset when $f_s=20$. Our experiments also showed that both the *BERT* variants and *BoW* possessed good interpretability, which is important for medical applications of coding models.

**Conclusions:**

This study shed light on building promising machine learning models for automated ICD coding by revealing the most effective feature extraction methods. Concretely, our results indicated that fine-tuning the whole network of the *BERT* variants was the optimal method for tasks covering only frequent codes, especially codes that represented unspecified diseases, while *BoW* was the best for tasks involving both frequent and infrequent codes. The frequency threshold where the best-performing method varied differed between different datasets due to factors like language and codeset.

**Supplementary Information:**

The online version contains supplementary material available at 10.1186/s12911-022-01753-5.

## Background

During patients’ visits at hospitals, rich text data is generated, such as diagnoses from health professionals. An important task is to assign the codes from the International Classification of Diseases (ICD) system to the text, with each code representing a disease or procedure. The coding task serves as basis for a wide range of applications, including reimbursement, epidemiological studies and health service research. At present, the task is mainly accomplished by clinical coders who are trained to grasp coding rules, yet manual coding is time-consuming and prone to errors, promoting automated ICD coding via machine learning to be a hot topic.

Using machine learning to fulfill automated ICD coding basically comprises two phases, feature extraction and classifier building. Feature extraction is crucially important, as it plays the role of a bridge between raw text and classifiers, and should extract useful features from raw text as many as possible. At present, there are three typical feature extraction methods, namely bag-of-words (*BoW*), word2vec (*W2V*) and large pre-trained natural language processing (*NLP*) models. *BoW* is widely used in traditional machine learning. It treats text as a collection of words without strict orders, and ignores complicated semantic and syntactic information. *W2V* was introduced by Mikolov et al. [1], and has been adopted in a great many studies. Splitting a training corpus into windows of text, *W2V* uses context words to predict central words (or vice versa), through which word embeddings for corresponding vocabulary can be learned. Compared with *BoW*, *W2V* is capable of guiding word embeddings to embody semantic and syntactic information in dense real-valued low-dimensional vectors. Large pre-trained *NLP* models gain much attention over recent years, the key point underlying which is first mining knowledge from large corpora with complicated neural networks, and then transferring the knowledge to downstream tasks to improve their performances. The most representative large pre-trained model is *BERT*, which was trained on large corpora from various fields and has been proven quite useful over many *NLP* tasks [2]. In comparison to *W2V*, models like *BERT* can learn far more language semantics and domain knowledge.

Existing studies relating to automated ICD coding can be categorized into two streams. The first is from medical field [3–14]. These studies focus on developing applicable models for a subset of ICD codes based on private datasets. *BoW* is adopted as the feature extraction method mostly, and conventional classifiers or similarity-based methods are commonly used to accomplish automated coding. In specific, using *BoW* and support vector machine (*SVM*), Karimi et al. (2017) auto-assigned 16 codes to radiology reports and resulted in a *Micro-F*1 of over 80% [8], Koopman et al. (2015) predicted 85 cancer related codes based on death certificates and reached a *F*1-score of 70% [6], and Kaur and Ginige (2018) automatically allocated two codes relating to respiratory and gastrointestinal systems and achieved a *F*1-score of 91.4% [7]. Applying *BoW* on unstructured clinical notes, Elyne et al. (2016) concluded that unstructured and structured data were complementary in predicting codes covering several medical specialties [14]. To the best of our knowledge, *W2V* and large pretrained *NLP* models, which hold advantages over *BoW* in analyzing syntax and semantics, have not been commonly used yet.

The second is from computer science [15–24], where most studies use *W2V* and deep learning neural networks as feature extraction methods, and mainly target on developing models on large public datasets, such as MIMIC-III [25–27]. For instance, Mullenbach et al. (2018) proposed a network named CAML, which consists of a convolutional layer and a label attention layer [18]. Lately, some studies are keen on pretraining *BERT*-like architectures on large medical corpora, with the purpose of making the model more fitted for medical missions. One example is *BioBert*, which was pretrained on PubMed corpora and confirmed effective in dealing with tasks such as ICD coding [28]. Although having adopted more advanced feature extraction methods, the metrics reported by these studies are generally not high. For instance, the state-of-the-art *F*1-score for the full codes in MIMIC-III is currently below 60% [20].

For the purpose of application, this study targeted on comparing *BoW*, *W2V* and *BERT* variants on auto-assigning ICD codes to medical records. Like some related studies [7, 8], the scale of the datasets in this study is limited, therefore we used logistic regression (*LR*) and *SVM* instead of deep learning models as classifiers. We designed coding tasks with different code frequency thresholds. In general, a higher threshold means more frequent codes to predict and thus a less complex task. Our goal is to uncover which feature extraction method is most effective, and whether the most effective one varies across tasks at different complex levels. Achieving the goal can be of help in building promising coding models and assisting coding practice.

## Methods

This section first briefly describes the feature extraction methods, classifiers and evaluation metrics used in this study, and then gives details of our methodology.

### Feature extraction methods

#### Bag-of-words

*BoW* is widely used in traditional machine learning. The model treats text as a collection of words (or $n-grams$) without strict orders, and ignores complicated semantic and syntactic information. To calculate weights of words in a document, the term frequency-inverse document frequency ($tf-idf$) method is mostly adopted. According to $tf-idf$, given a corpus *D* containing *N* documents, the weight of word $w_i$ in document $d_j$ is:

1 $$\begin{aligned} {w_{i,j}} = t{f_{i,j}} \times \log \left(\frac{N}{{d{f_i}}}\right) \end{aligned}$$

where $tf_{i,j}$ is the term frequency of $w_i$ in $d_j$ and $df_i$ is the number of documents that mention $w_i$. As the equation indicates, words occurring more in $d_j$ and less in *D* are considered more representative of $d_j$ and given higher weights. Features from *BoW* are generally high-dimensional and sparse.

#### Word2vec

**Fig. 1:**
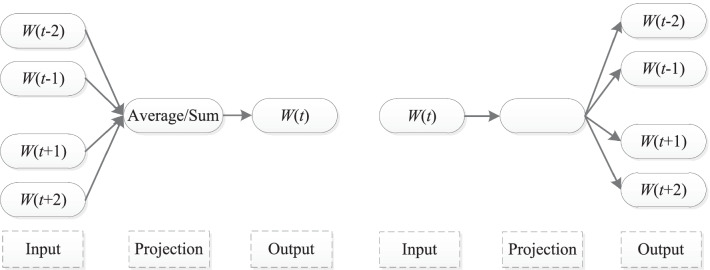
Word2vec. Left: *CBOW*. Right: *skip-gram*

Since proposed by Mikolov et al. [1], *W2V* has been widely used in both traditional machine learning and deep learning studies. The method involves two alternative models, continuous bag-of-words (*CBOW*) and *skip-gram*, both of which are simple multiple-layer perceptron structures, as shown in Fig. 1.

As preprocessing, *W2V* transforms a training corpus into text windows of pre-defined size, and randomly initializes word embeddings for corresponding vocabulary. Given a text window during training, *CBOW* uses context words to predict the central word, while skip-gram uses the central word to predict context words. Training loss is assessed by cross entropy and word embeddings are gradually adjusted during backpropagation. After enough training, the embeddings tend to converge and are ready for downstream tasks.

Compared with *BoW* that only utilizes frequency information, *W2V* is capable of extracting abstract semantic and syntactic features which are dense, real-valued and low-dimensional.

#### Large pretrained *NLP* models

**Fig. 2:**
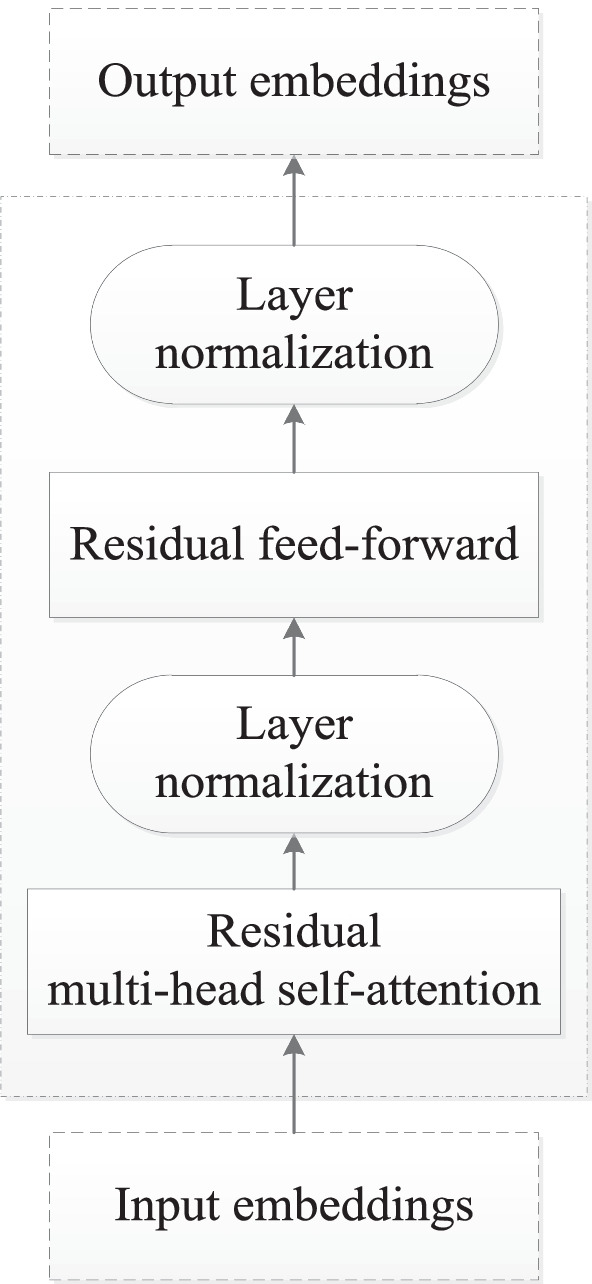
The unit layer of the encoder of Transformer

The basic idea underlying large pre-trained *NLP* models is employing complex neural networks to mine knowledge from large corpora first, and then transferring the knowledge to downstream tasks to improve their performances. *BERT* is a typical such model [2], which has received much attention lately [29–34]. The neural network of *BERT*^1^ is a 12-layer encoder of Transformer [35], where each layer consists of a residual multi-head self-attention layer and a residual feed forward layer, both of which are followed by layer normalization, as shown in Fig. 2. Next sentence prediction (*NSP*) and masked language modeling (*MLM*) are used as learning tasks. After trained on a large corpus from various fields, *BERT* has been proven very useful in fulfilling a wide range of *NLP* tasks [36, 37]. Some studies proposed some variants of *BERT*, one of which is *RoBERTa* [30], which uses the same network as *BERT*, but was trained with improved procedures such as larger batches and more steps. Note that *W2V* provides word embeddings that are static and context-agnostic once trained. In contrast, *BERT* and its variants play the role of sentence encoders, as they encode sequences of tokens and provided embeddings for tokens by taking contextual information in the sequences into account. This can be aiding in handling ambiguity in text and extracting more informative features for down-stream tasks.

### Classifiers

#### Logistic regression

*LR* is widely used as a baseline classifier because of its simplicity and high efficiency [38]. Given a binary dependent variable *y* and *m* predictors $x=\{x_1,x_2,\ldots ,x_m\}$, the model can be expressed as:

2 $$\begin{aligned} p(y = 1) = \frac{1}{{1 + {e^{ - (\alpha + {\beta ^T}x)}}}} \end{aligned}$$

where $\alpha$ and $\beta$ are intercept and coefficients respectively, and can be estimated via maximum likelihood estimation. Provided with *n* observations$(x^i,y^i )$ $(1\le i\le n)$, the log likelihood of *LR* regularized with *L*2 norm is:

3 $$\begin{aligned} l(\alpha , \beta ) = \sum \limits _{i = 1}^n {\ln p({y^i}|{x^i},\alpha ,\beta )} - \frac{\lambda }{2}\left({\alpha ^2} + \sum \limits _{j = 1}^m {\beta _j^2} \right) \end{aligned}$$

where $\lambda$ is a positive penalty factor. Maximizing $l(\alpha , \beta )$ using gradient descent methods leads to optimal parameters $\alpha ^\star$ and $\beta ^\star$ that fit training data best. As we focused on comparing different feature extraction methods, we did not tune $\lambda$ comprehensively. Choosing $\lambda$ from 1, $\frac{1}{2}$, $\frac{1}{3}$, $\frac{1}{4}$, $\frac{1}{5}$, we implemented training and tests on some randomly sampled data, and found $\lambda =\frac{1}{5}$ generally led to better results. Hence $\lambda =\frac{1}{5}$ was used throughout the study.

#### Support vector machine

*SVM* is one of the most successful conventional classifiers, due to its capability of handling a large number of features and simultaneously being memory efficient [39], and has been adopted in many studies for automated ICD coding [5–8]. Given a data set $D=\{(x_i,y_i)|y_i=1/-1,1\le i\le n\}$ where $x_i$ represents feature values, $y_i$ is a class label and *n* indicates data volume, *SVM* aims at searching for the hyperplane with the largest margin *P*: $wx+b=0$ that separates ${D_0} = \{ ({x_i},{y_i})|{y_i} = - 1\}$ from ${D_1} = \{ ({x_j},{y_j})|{y_j} = 1\}$. *w* and *b* are usually calculated by solving the following optimization problem:

4 $$\begin{aligned} \mathop {\min }\limits _{w,b,{\xi _i}} \{ \frac{1}{2}{w^T}w + \frac{C}{2}\sum \limits _{i = 1}^n {\xi _i^2} \} \end{aligned}$$

Subject to:

5 $$\begin{aligned} \left\{ \begin{array}{l} {y_i}({w^T}{\phi (x_i)} + b) \ge 1 - {\xi _i}\\ {\xi _i} \ge 0,\mathrm{{ }}1 \le i \le n \end{array} \right. \end{aligned}$$

$\xi =\{ {\xi _i}:1 \le i \le n\}$ are called slack variables, standing for the tolerance of *SVM* for misclassifications. *C* is a positive penalty factor on the slack variables. A larger *C* generally leads to higher training accuracy, yet puts the model under the risk of overfitting. $\phi (x_i)$ is a transformation function which transforms data *x* into a new space, with the purpose of increasing the chance of separating data belonging to different classes which can not be separated in the original space. Kernel functions *K*(*x*, *y*), which satisfies: $K(x, y)=\phi (x)\cdot \phi (y)$, is introduced to simplify the transformation calculations. Commonly used kernel functions include linear function and radial basis function. In this study, linear kernel function was used. Using the same method of selecting $\lambda$ for *LR*, we chose $C=\frac{1}{5}$ for *SVM* in all of the experiments.

### Data

#### Basic introduction

**Fuwai dataset**

Fuwai Hospital is a Chinese hospital featured in treating cardiovascular diseases. With the approval from the Ethics Committee at Fuwai Hospital, we obtained a dataset lasting from January 2019 to February 2019, which includes no identifiable personal information. The dataset contains 6947 records, in which each record consists of a textual diagnosis summary for a patient and a list of codes, which are the Chinese version of standard ICD-10 diagnosis codes. Totally, the dataset involves 1532 unique codes. As preprocessing, we cleaned the diagnosis summaries by removing all numbers and symbols except for ‘[’ and ‘]’^2^ , and used *Jieba*^3^ package to cut the cleaned text into words. To obtain medical terms more precisely, we loaded a Chinese medical vocabulary from *Sogou*^4^ into *Jieba*, which contains 90,047 terms relating to diagnoses, medicine and so on.

**CodiEsp dataset**

CodiEsp dataset [40] is a public dataset released by the CLEF eHealth 2020 conference^5^. The dataset contains 1000 Spanish clinical records, and provides both Spanish and English textual diagnosis summaries. In this study, the Engish version was used. A list of gold standard CIE-10 diagnosis codes, which are the Spanish version of ICD-10 diagnosis codes, were assign to each record. 2557 unique codes appear in the dataset. We deleted all symbols, numbers and stop words in the records at the preprocessing stage.

#### Descriptive analysis

Descriptive statistics of the datasets are listed in Table 1. For Fuwai data, we additionally summarized the character-level statistics.

**Table 1:** Descriptive statistics of the datasets

	Fuwai			CodiEsp	
	Word	Character	Code	Word	Code
Token size	691,418	1,557,769	44,366	161,078	11,158
Vocabulary size	9130	1768	1532	14,885	2557
Average length	99.5	224.2	6.4	161.1	11.2

**Fig. 3:**
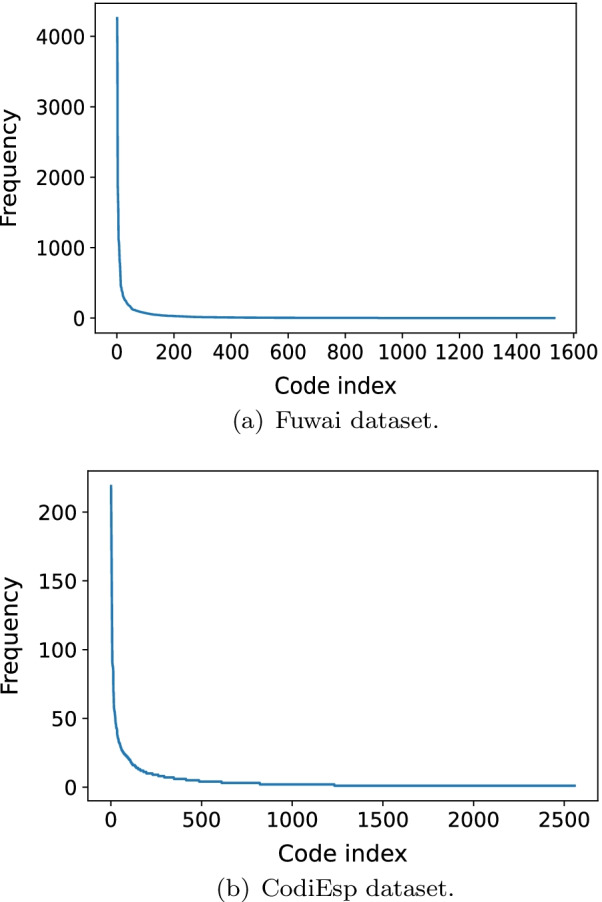
The distribution of code frequencies in the datasets

**Fig. 4:**
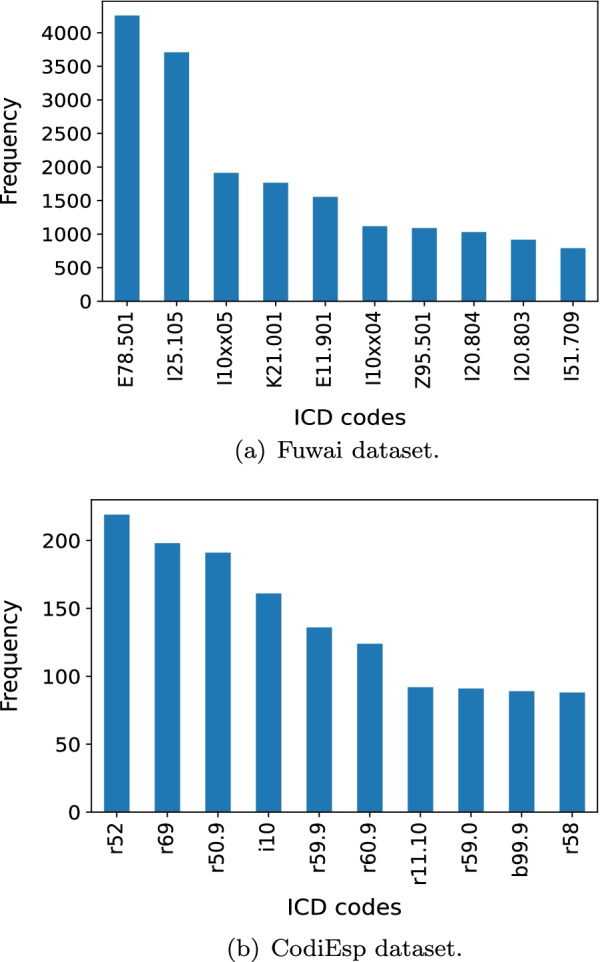
The most frequent 10 codes in the datasets

For each dataset, we ranked the codes by their frequencies in descending order, and plotted the frequencies against the rankings (Fig. 3). Apparently, the distributions of the code frequencies in both datasets follow long-tail distribution. Figure 4 shows the 10 most frequent codes and their frequencies regarding each of the datasets.

Codes with few records would result in overfitting when training classifiers. Therefore, we selected a subset of codes to predict by setting a code frequency threshold $f_s$. Intuitively, a smaller $f_s$ means more infrequent codes to predict and thus a more complex coding task. To find out whether the best feature extraction method varies across tasks at different complex levels, we experimented with different thresholds on each of the datasets. Under each threshold, we only used data relating to at least one of qualified codes. 80% of selected data was for training and the rest was for test.

### Evaluation metrics

In accordance with related studies [6, 7, 16, 18], we mainly used *F*1-score and *AUC* to assess coding performance. *Micro-F*1, *Macro-F*1, *Micro-AUC* and *Macro-AUC* were used as specific metrics. A micro metric corresponds to the hypothetical single code that integrates all individual codes, while a macro metric is the mean of metrics for each individual code. Micro metrics place more weights on codes with more records. As a comparison, macro metrics treat each code equally. In automated ICD coding where codes are most likely to distribute disproportionately, micro metrics, especially *Micro-F*1, are generally given more attention.

*F*1-score and *AUC* regarding a single code are described as follows.

#### *F*1

Assume there are a set of records among which $t_1$ are in class 1 indicating a code is assigned. After feeding the records into a trained model, $p_1$ are tagged with 1, within which $tp_1$ are correctly tagged. Then the *F*1-score of the coding performance is:

6 $$\begin{aligned} F1 = \frac{{2 \times Precision \times Recall}}{{Precision + Recall}} \end{aligned}$$

where $$Precision=tp_1/p_1$$ and $$Recall=tp_1/t_1$$.

#### *AUC*

*AUC* stands for area under the curve, where the curve is receiver operating characteristic (*ROC*) curve. Many classification models, including *LR* and *SVM*, finally output probabilities that records belong to class 1, instead of directly 1 or 0. Therefore, a calibration threshold needs to be used to transform the probabilities to classes . With *ROC*, numerous such thresholds are firstly selected, and true positive rate (*TPR*) and false positive rate (*FPR*) under each threshold are computed. Then the *TPR*s are plotted against the *FPR*s, resulting in the curve. A larger *AUC* indicates a model with better performance.

### Method design

**Fig. 5:**
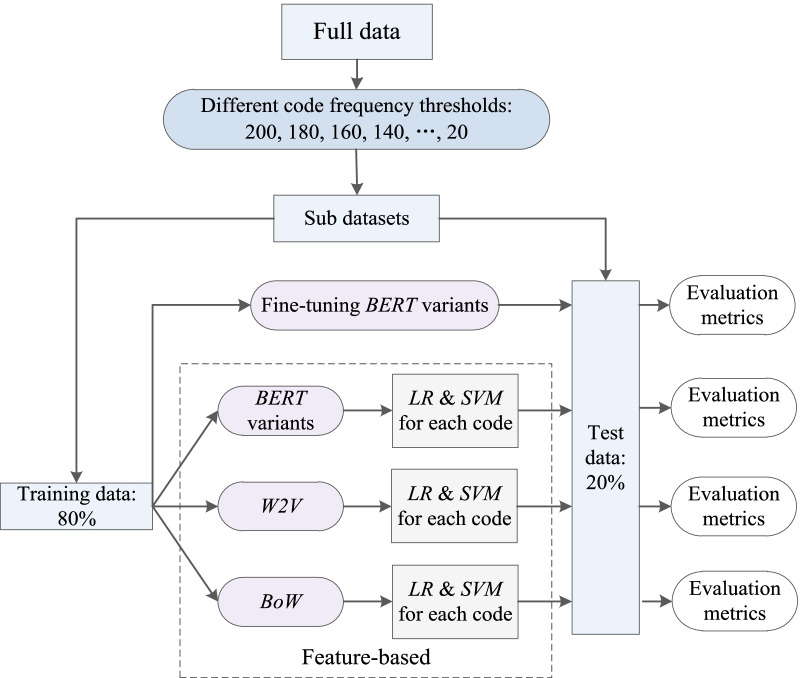
The framework of our methodology

Figure 5 depicts the framework of our methodology. We designed coding tasks with different code frequency thresholds. In terms of each coding task, we accomplished code prediction by both feature-based methods and fine-tuning *BERT* variants, and evaluated coding performance on test data. Details of the feature extraction methods are given below.

#### BoW

We extracted features via unigram, unigram+bigram, unigram+bigram+trigram separately. As there are generally tens of thousands of features resulted from each combination, we applied the filter method with Chi-square test to select most significant 1,000 features during training. For each feature, we calculated its significance with respect to each individual code, and took the maximum significance as the final metric for ranking the feature. Related experiments were finished using *Sklearn* package [41].

#### W2V

Using *gensim* package [42], we trained both word and character embeddings based on Fuwai dataset, and word embeddings based on CodiEsp dataset. Parameters used during training and their descriptions are listed in Table 2.

**Table 2:** Parameters for training *W2V* embeddings

Parameters	Descriptions	For Fuwai dataset	For CodiEsp dataset
*sg*	Whether Skip-gram is used.	1	1
*size*	The dimension of resulting embeddings.	256	128
*window*	The length of a text window.	5	5
*iter*	Training on data for how many iterations.	5	5
*min_count*	Discarding words/characters appearing less than how many times.	3	3

We used average pooling to obtain the embedding of a textual record. Specifically, we looked up embeddings of all words (characters) in a record and took the mean at all dimensions as word-level (character-level) record embedding. For Fuwai dataset, we respectively adopted record embeddings at character-level and word-level, and also used the concatenation of both kinds of embeddings in the experiments.

#### BERT variants

For Fuwai dataset, we used a Chinese pre-trained model named *RoBERTa-Mini* [2, 30, 43, 44], which has 4 layers of transformer encoder units and represents characters with embeddings of 256 dimensions in each layer. Note that in the Chinese context, pre-trained *NLP* models are generally character-based rather than word-based, due to tremendous size of Chinese word vocabulary. Albeit recently there are some attempts at word-based models like *WoBert* [45], they only cover a vocabulary of limited size, and would encounter severe out-of-vocabulary problems when used in medical studies.

For CodiEsp dataset, we used a English pre-pretrained model named *BERT-mini* [46, 47], which has the same network structure as *RoBERTa-Mini*.

As both models can handle input sequences up to 512 tokens, medical records longer than 512 tokens were truncated in our experiments, while those shorter than 512 tokens were padded with meaningless tokens. For Fuwai dataset, among all the coding tasks, at most 4.6% of more than 6,000 records were truncated. The deleted content was mostly detailed symptom descriptions. Therefore, the truncation imposed little influence on the coding performance of *RoBERTa-Mini*. As for CodiEsp dataset, no clinical records need to be truncated.

Regarding both *BERT* variants, the outputs from the last layer were used, which mainly consists of two parts. One is a feature vector for the [CLS] token, which is automatically added on the beginning of each input sequence for the *NSP* task. The other is a embedding matrix where columns correspond to input tokens. Accordingly, we adopted two methods to achieve automated coding. The first is adding a fully connected layer above the feature vector, which contains the same number of neurons as that of target codes. Sigmoid was used as the activation function. During training we fine-tuned the whole network and only the top fully connected layer respectively. *Adam* was employed as the optimization algorithm, with batch size set to 32 and binary cross entropy as the loss function. Considering the random issue induced by operations such as parameter initialization and data split, we ran 5 rounds of training and tests when using the fine-tuning method. Within each round, different random seeds were used to split data. The mean of metrics from all the rounds were reported finally. The second method is using the embedding matrix as token features to train *LR* and *SVM*, with columns corresponding to padded tokens excluded. Average-pooling was used to generate record embeddings. Besides using the embedding matrix directly, we also experimented with concatenating the embeddings from the *BERT* variants and *W2V*.

## Results

### Frequent codes only

We started with fixing the code frequency threshold $f_s$ as relatively high values, in which situation each chosen code occurs frequently in the datasets. In specific, for Fuwai data, we set $f_s$ as 200, and 37 codes meet the standard, whose occurrences account for 61.6% of the total code occurrences. 6,398 records were selected. For CodiEsp dataset, we set $f_s$ as 180, and 3 codes meet the standard, whose occurrences account for 5.4% of the total code occurrences. 473 clinical records were selected. Coding results for the datasets are listed in Tables 3 and 4, respectively. The largest *Micro-F*1 for each feature extraction method is shown in bold, and the largest globally is marked in underline.

**Table 3:** Coding results for Fuwai dataset with $f_s = 200$

Feature extraction & classifiers	*Macro-F1* (%)	*Micro-F1* (%)	*Macro-AUC* (%)	*Micro-AUC (%)*
*BoW*				
* LR_uni*	84.44	91.54	88.58	93.75
* SVM_uni*	84.69	91.78	89.27	94.10
* LR_uni_bi*	84.83	**92.27**	89.08	94.41
* SVM_uni_bi*	83.02	91.57	88.23	93.93
* LR_uni_bi_tri*	83.01	91.50	88.00	93.88
* SVM_uni_bi_tri*	78.21	89.45	85.20	92.19
*W2V*				
* LR_word*	53.14	75.07	71.60	82.05
* SVM_word*	35.73	64.92	64.09	75.10
* LR_char*	48.03	70.54	68.77	79.04
* SVM_char*	26.30	58.86	60.37	71.64
* LR_comb*	61.73	**80.27**	75.75	85.47
* SVM_comb*	46.26	73.68	69.17	80.51
*RoBERTa_embeddings*				
* LR_char*	64.56	78.59	77.90	85.51
* SVM_char*	51.30	75.24	71.86	82.45
* LR_comb*	72.41	**84.20**	82.23	89.07
* SVM_comb*	64.25	81.41	77.57	86.44
*RoBERTa_finetune*				
* top_layer*	4.31	40.59	69.56	80.32
* whole*	83.39	93.87	98.65	99.55

For *BoW*, *_uni*, *_uni_bi* and *_uni_bi_tri* mean unigram, unigram+bigram and unigram+bigram+trigram respectively. For *W2V*, *_comb* means concatenating character and word embeddings, while *_char* (*_word*) means merely character (word) embeddings. For *RoBERTa_embeddings*, *_char* means merely the *RoBERTa-Mini* embeddings, and *_comb* means concatenating the *RoBERTa-Mini* embeddings and *W2V* word embbeddings. For *RoBERTa_finetune*, *whole* and *top_layer* mean fine-tuning the whole network and only the top fully connected layer respectively

**Table 4:** Coding results for CodiEsp dataset with $f_s = 180$

Feature extraction & classifiers	*Macro-F1* (%)	*Micro-F1* (%)	*Macro-AUC* (%)	*Micro-AUC* (%)
*BoW*				
* LR_uni*	63.55	63.68	70.44	70.04
* SVM_uni*	70.68	70.34	75.13	74.45
* LR_uni_bi*	63.93	63.85	72.36	71.08
* SVM_uni_bi*	72.46	**72.27**	77.22	75.95
* LR_uni_bi_tri*	62.41	62.26	71.39	69.97
* SVM_uni_bi_tri*	69.48	69.26	75.39	73.90
*W2V*				
* LR_word*	56.07	56.07	64.39	64.62
* SVM_word*	59.52	**59.63**	66.86	67.11
*BERT_embeddings*				
* LR_word*	64.00	**63.90**	69.33	68.61
* SVM_word*	59.15	59.02	64.29	64.11
* LR_comb*	61.26	60.91	66.32	65.85
* SVM_comb*	62.52	62.45	67.68	67.73
*BERT_finetune*				
* top_layer*	17.21	22.19	48.79	49.40
* whole*	85.32	85.41	91.44	92.82

Aside from *BERT_embeddings*, the suffixes have the same meanings as those in Table 3. For *BERT_embeddings*, *_word* means merely the *BERT-mini* embeddings, and *_comb* means concatenating the *BERT-mini* embeddings and *W2V* word embbeddings

Regarding the classifiers, for Fuwai dataset, *LR* mostly performed better than *SVM* over all the metrics using same features, whereas for CodiEsp dataset, *SVM* mostly outperformed *LR* over all the metrics given same features. The result held across experiments with different $f_s$ for both datasets.

Focusing on the feature extraction methods for Fuwai dataset, *RoBERTa-Mini* with the whole network fine-tuned achieved the best results regarding all the metrics except for *Macro-F*1, and reached a *Micro-F*1 of 93.87%, a *Micro-Precision* of 95.38%, and a *Micro-Recall* of 92.43%. As a dramatic comparison, *RoBERTa-Mini* with only the top layer fine-tuned performed quite poorly.

As for the other methods, *BoW* led to more promising results, with a *Micro-F*1 of 92.27%, a *Micro-Precision* of 95.35%, and a *Micro-Recall* of 89.39%. In regards of the embedding methods, using word and character embeddings together outperformed using either one. Specifically, concatenating the *RoBERTa-Mini* character embeddings and *W2V* word embeddings was better than the other options, reaching a *Micro-F*1 of 84.2%, a *Micro-Precision* of 89.7%, and a *Micro-Recall* of 79.34%.

Similar conclusions can be drawn for CodiEsp dataset. *BERT-mini* with the whole network fine-tuned performed best, reaching a *Micro-F*1 of 85.41%, a *Micro-Precision* of 85.4%, and a *Micro-Recall* of 85.6%. *BoW* followed, resulting in a *Micro-F*1 of 72.27%, a *Micro-Precision* of 77.48% and a *Micro-Recall* of 67.72%. Regarding the embedding methods, solely using the word embeddings from *BERT-mini* performed better than the other options, leading to a *Micro-F*1 of 63.9%.

### Considering both frequent and infrequent codes

We chose relatively low $f_s$ in this section, in order to find out the most effective feature extraction methods when analyzing both frequent and infrequent codes.

Specifically, $f_s = 20$ was used for both datasets. For Fuwai dataset, 248 codes and 6,906 records were chosen. The occurrences of the code subset account for 90.1% of the total code occurrences. For CodiEsp dataset, 106 codes and 931 records were selected, with 41.4% of the total code occurrences covered. Coding results for the datasets are separately listed in Tables 5 and 6.

**Table 5:** Coding results for Fuwai dataset with $f_s = 20$

Feature extraction & classifiers	*Macro-F*1 (%)	*Micro-F*1 (%)	*Macro-AUC* (%)	*Micro-AUC* (%)
*BoW*				
* LR_uni*	52.95	82.99	71.82	86.88
* SVM_uni*	47.41	82.70	70.46	86.73
* LR_uni_bi*	46.25	80.79	69.10	85.48
* SVM_uni_bi*	37.70	79.07	66.11	84.13
* LR_uni_bi_tri*	39.12	72.85	65.93	80.49
* SVM_uni_bi_tri*	27.24	67.62	61.68	76.77
*W2V*				
* LR_word*	22.81	63.29	58.79	74.85
* SVM_word*	12.74	53.46	55.07	68.99
* LR_char*	19.16	58.43	57.17	72.09
* SVM_char*	8.16	45.92	53.19	65.40
* LR_comb*	29.08	**69.02**	61.32	78.13
* SVM_comb*	16.92	61.84	56.97	73.39
*RoBERTa_embeddings*				
* LR_char*	34.75	69.03	63.89	79.25
* SVM_char*	23.41	64.75	59.58	75.74
* LR_comb*	39.44	74.32	66.00	82.17
* SVM_comb*	29.64	**70.59**	62.16	79.01
*RoBERTa_finetune*				
* top_layer*	0.67	31.06	62.83	84.21
* whole*	2.43	**41.25**	75.00	90.26

**Table 6:** Coding results for CodiEsp dataset with $f_s = 20$

Feature extraction & classifiers	*Macro-F*1 (%)	*Micro-F*1 (%)	*Macro-AUC* (%)	*Micro-AUC* (%)
*BoW*				
* LR_uni*	5.96	13.81	51.81	53.72
* SVM_uni*	24.06	39.13	58.61	62.61
* LR_uni_bi*	2.42	6.29	50.70	51.62
* SVM_uni_bi*	12.79	23.56	54.39	56.75
* LR_uni_bi_tri*	1.55	4.04	50.43	51.03
* SVM_uni_bi_tri*	8.14	14.76	52.70	54.00
*W2V*				
* LR_word*	0.57	**2.31**	50.15	50.57
* SVM_word*	0.00	0.00	50.00	50.00
*BERT_embeddings*				
* LR_char*	15.81	22.77	55.42	57.28
* SVM_char*	15.34	21.39	56.00	57.55
* LR_comb*	17.71	**26.45**	56.25	58.78
* SVM_comb*	18.24	25.75	57.43	59.68
*BERT_finetune*				
* top_layer*	0.01	0.04	52.70	65.02
* whole*	1.72	**6.71**	68.40	74.87

The results for both datasets diffed a lot from those in the last subsection. The methods of fine-tuning the *BERT* variants performed poorly. *BoW* became the best in terms of *Micro-F*1. For Fuwai dataset, it reached a *Micro-F*1 of 82.99%, a *Micro-Precision* of 94.69%, and a *Micro-Recall* of 73.86%. For CodiEsp dataset, it led to a *Micro-F*1 of 39.13%, a *Micro-Precision* of 84.78%, and a *Micro-Recall* of 25.43%.

Regarding the embedding methods, using the embeddings from the *BERT* variants and *W2V* together was the best choice for both datasets.

### Results with multiple code frequency thresholds

**Fig. 6:**
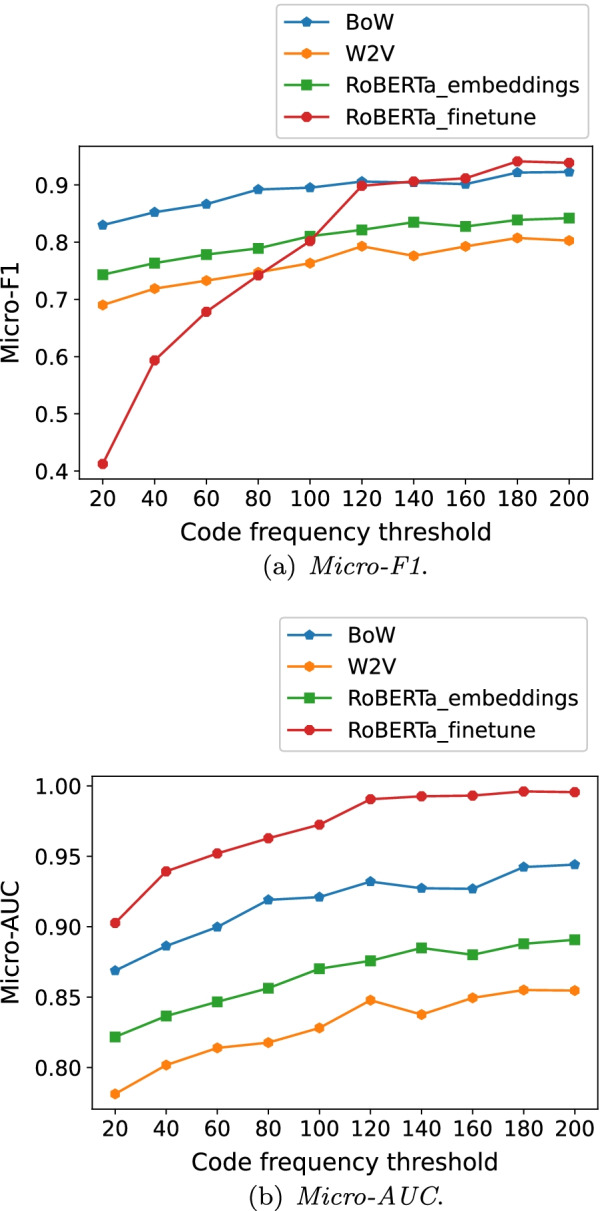
The metrics for the feature extraction methods on Fuwai dataset

**Fig. 7:**
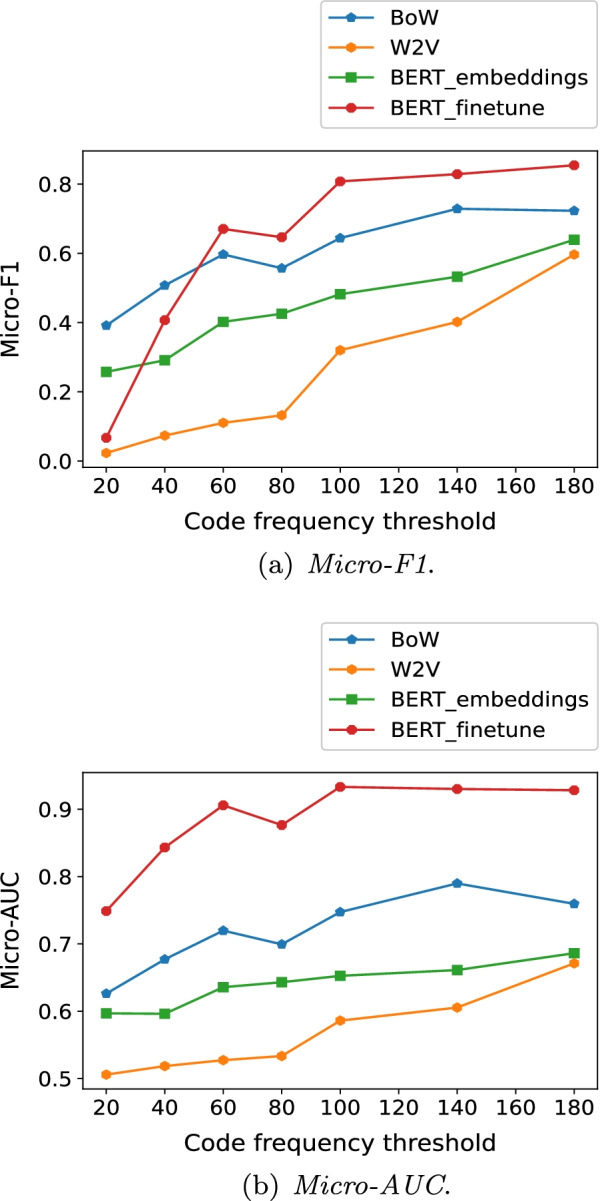
The metrics for the feature extraction methods on CodiEsp dataset

We intended to uncover more details about how the best feature extraction method varied with respect to code frequency thresholds. Accordingly, we let $f_s$ change between (20, 200) for Fuwai dataset and increased it by 20 each time. For CodiEsp dataset, we let $f_s$ take 40, 60, 80, 100, and 140 respectively^6^. For each of the thresholds, the number of the qualified codes and selected records, and the proportion of the total code occurrences covered by the qualified codes are given in Additional file 1. Figures 6 and 7 display the best *Micro-F*1 and *Micro-AUC* under each of the feature selection methods in relation to the multiple $f_s$.

Interestingly, fine-tuning the whole network of the *BERT* variants consistently led to the highest *Micro-AUC* for both datasets. As the definition indicates, a higher *AUC* generally means higher *TPR* and 1-*FPR*, in other words, higher *Recall* for both positive and negative cases given multiple calibration thresholds, and it does not directly relate to *Precision* for positive cases. However, In the ICD coding task, both *Precision* and *Recall* for positive cases are quite important in coding practice, and can be captured by *F*1-score. Hence, we placed more weights on *Micro-F*1 when evaluating the feature extraction methods^7^.

Focusing on Fuwai dataset, the point where $f_s = 140$ can be observed as a turning point. When $f_s$ was equal to or greater than 140, *RoBERTa-Mini* with the whole network fine-tuned resulted in the highest *Micro-F*1. When $f_s$ was lower than 140, *BoW*, mostly used together with *LR*, performed best.

As for CodiEsp dataset, there also exists a turning point, the one where $f_s = 60$. When $f_s$ equaled or exceeded 60, fine-tuning the whole network of *BERT-mini* was most effective. Once $f_s$ fell below 60, *BoW* in conjunction with *SVM* consistently led to the highest *Micro-F*1.

Regarding the embedding methods on both datasets, using the embeddings from the *BERT* variants and *W2V* together generally achieved higher *Micro-F*1, in comparison to using merely the embeddings from the *BERT* variants or *W2V*.

Note that the advantage of the *BERT* variants over *BoW* in terms of *Micro-F*1 was more obvious and lasted for a wider range on CodiEsp dataset than that on Fuwai dataset. Besides factors like the difference in data volume and pretraining details of the *BERT* variants, the main reason could be as follows. In Fuwai dataset, most codes stand for specified diseases, such as E78.501 (hyperlipemia) and I25.105 (coronary atherosclerotic heart disease). There are some unique $n-grams$ features quite indicative of these codes, such as ’atherosclerotic’ for I25.105, and these features could be captured effectively by *BoW* when the feature selection was implemented, leading to competitive coding performance of the *BoW* methods on the frequent or infrequent codes. As a contrast, in CodiEsp dataset, many codes stand for unspecified diseases, such as r52 (pain, not elsewhere classified) and r69 (illness unspecified). Among the 20 most frequent codes, 13 are such kind of codes. The uncertainty behind these codes might make *BoW* struggle in capturing informative $n-grams$ features for predicting the codes. However, due to the capability of handling ambiguity in text, the *BERT* variants could perform promisingly when extracting useful features for code assignment, as long as enough records for target codes are provided.

### Interpretability

The experiments above indicated that the *BERT* variants with the whole network fine-tuned was the optimal feature extraction method when assigning merely frequent codes, while *BoW* became most effective when predicting both frequent and infrequent codes. This section shows that both the *BERT* variants and *BoW* possess good interpretability in automated coding, which is important for medical applications of coding models.

As for the *BERT* variants, the attention weights from its top layer give hints on how the models allocate importance for input tokens. In specific, the feature vector for code prediction is the weighted average of all embeddings of input tokens from the top layer. Higher attention weights mean more important roles of corresponding tokens in computing the feature vector. By observing the distribution of the weights, we can gain a straightforward view of what are key tokens, and whether those tokens are useful after referring to target codes.

Both *BERT* variants in this study adopt 4-head self-attention mechanism, indicating that there are four groups of attention weights for input tokens. We used the largest weight for each token as the metric of importance and ranked all tokens in descending order according to the metric.

In terms of *BoW*, key features selected by the filter method are shared by all inputs, hence the interpretability can be achieved by analysing whether the key features are informative or not in relation to target codes.

For Fuwai dataset, we defined $Pro\_K$ and $Pro\_N$ to quantify the explainability of *RoBERTa-Mini* and *BoW*, respectively.

Given a diagnosis summary, we computed the number of characters that appear in both top *K* key characters from *RoBERTa-Mini* and corresponding code labels, and divided the number by *K*. The result can be seen as a metric for explainability at single record level. $Pro\_K$ equaled the mean over such metrics from all test records.

We calculated the number of words that appear in both top *N* key words^8^ from Chi-square test and corresponding code labels, and divided the number by *N* to gain $Pro\_N$.

Fixing $f_s$ as 200, we report $Pro\_K$ for several *K* based on a randomly selected round of experiment in Table 7, and $Pro\_N$ for several *N* in Table 8.

**Table 7:** $Pro\_K$ for interpreting the code assignment by *RoBERTa-Mini*

*K*	1	2	3	4	5
*Pro_K*	73.2%	74.2%	74.7%	75.2%	75.0%

**Table 8:** $Pro\_N$ for interpreting *BoW*

*N*	10	20	30	40	50
*Pro_N*	80.0%	80.0%	83.0%	80.0%	76.0%

The tables show that both *RoBERTa-Mini* and *BoW* precisely located useful information for assigning the target codes.

In terms of CodiEsp dataset, $Pro\_K$ and $Pro\_N$ do not fit for *BERT-mini* and *BoW*. As mentioned above, many codes in the dataset represent unspecified diseases. As a result, many key words selected by feature extraction methods might not appear in such code labels, even if they are predictive of the codes. Hence, we give two examples to show the interpretability of *BERT-mini* and *BoW* below.

Fixing $f_s$ as 180, we list the top 10 key words selected through *BoW* and the 3 target codes in Table 10. For *BERT-mini*, we randomly display a clinical record and its ICD codes in Table 9, with the top 5 key words shown in bold.

**Table 9:** A clinical record for interpreting the code assignment by *BERT-mini*

Case description	ICD code
year male patient evaluated pain grade iii obliterating arteriopathy involvement limbs received analgesic treatment **durogesic** matrix months acceptable pain control vas **rescue** medication paracetamol maximum daily **pain** unit emergency visit days increased pain threshold agitation nervousness picture occurs result bedside doctor medication prescribed transdermal fentanyl generic requiring rescue paracetamol **increasing** vas pain relief anamnesis patient prescribed durogesic matrix patient reviewed weeks presents pain relief vas disappearing nervousness presented months visit patient continues durogesic matrix occasionally **paracetamol**	r52 (pain not elsewhere classified)

**Table 10:** 10 key words and the target ICD codes

Key words	Target codes
fever, disease, pain, antibiotic, drainage, crp, painful, leukocytosis, vas, pleural	r52(pain, not elsewhere classified), r69(illness unspecified), r50.9(Fever, unspecified)

The examples intuitively demonstrate that both *BERT-mini* and *BoW* identified valuable information to predict the target codes.

## Discussion

As expected, the lower the code frequency threshold, the more complex corresponding tasks, and the lower the performance metrics for all the feature extraction methods.

Experiments on both datasets suggested that the performance of the *BERT* variants changed dramatically across tasks at different complex levels. When handling frequent codes, the *BERT* variants reached the most promising results, and their advantage over other feature extraction methods was more obvious when handling codes representing unspecified diseases. When handling infrequent codes, the *BERT* variants performed poorly, probably because input tokens relating to the infrequent codes were rare and not sufficiently seen by the *BERT* variants, and as a consequence useful semantic representation for such tokens could not be learned.

*BoW* and the embedding methods were more stable compared with fine-tuning the *BERT* variants, among which *BoW* performed better, suggesting that *BoW* was more suited for coding tasks that covered both frequent and infrequent codes. The probable reason why *BoW* was relatively effective for infrequent codes was as follows. Combined with $tf-idf$ and the feature selection via Chi-square test, *BoW* could capture some rare words or phrases that were closely associated with infrequent codes.

The frequency threshold that indicates the change of the best-performing feature extraction method varied between different datasets. This could be attributable to many factors, such as language, data volume and the number of predicted codes, and we can not pinpoint a single one as the major cause.

Focusing on the embedding methods, using embeddings from both the *BERT* variants and *W2V* was the optimal choice in most cases.

This study faces some limitations. First, we only used text data following a number of related studies [4, 5, 8, 9]. However, as some research recorded [14, 23], both unstructured and structured data, such as various lab results, can help predict ICD codes. Whether using unstructured and structured data simultaneously would affect our conclusions need to be further verified.

Second, limited by the scale of the available datasets, we only employed traditional classifiers as many studies did [5–8]. These classifiers might not be capable of fully taking advantage of the information in the embeddings from *W2V* and the *BERT* variants, and this is probably why the embeddding methods performed not so well in our experiments. In the future, when datasets of larger scale are available, we will build sophisticated deep learning classifiers to check whether the embeddings would lead to more promising coding performance.

Third, currently, we merely experimented with a private Chinese dataset and a public Spanish dataset. According to related studies [10, 13], the portability of machine learning models for automated ICD coding might not be guaranteed. In the future, we will test the robustness of our conclusions by experimenting on more public datasets.

## Conclusion

This study aimed at comparing different feature extraction methods, namely *BoW*, *W2V* and *BERT* variants, when building applicable models for automated ICD coding. Our experiments demonstrated that the *BERT* variants with the whole network fine-tuned was optimal for coding tasks covering only frequent codes, especially codes representing unspecified diseases, and *BoW* turned into the best when coding tasks involved both frequent and infrequent codes. The frequency threshold at which the best feature extraction method changed varied across different datasets, probably because of factors like language and codeset. Besides, both the *BERT* variants and *BoW* possessed good interpretability. The conclusions can be of help in building effective coding models.

## Supplementary Information

**Additional file 1**. The numbers and occurrences of qualified codes with respect to the multiple code frequency thresholds. 

**Additional file 2**. The Micro-F1 of the BERT variants with different calibration thresholds.

## Data Availability

CodiEsp dataset can be found at: https://zenodo.org/record/3837305#.YYm_rWBBw2x. Fuwai dataset is not publicly available due to reasonable privacy and security concerns, and it is not easily redistributable to researchers other than those engaged in the research approved by the Ethics Committee at Fuwai Hospital.

## Abbreviations

CBOW: Continuous bag-of-words
ICD: International Classification of Diseases
AUC: Area under the ROC curve
ROC: Receiver operating characteristic
NLP: Natural language processing
LR: Logistic regression
SVM: Support vector machine
tf-idf: Term frequency-inverse document frequency
W2V: Word2vec
NSP: Next sentence prediction
MLM: Masked language modeling
CVD: Cardiovascular diseases
TPR: True positive rate
FPR: False positive rate

## Footnotes

1 *BERT* means the base model of *BERT* in this paper.

2 The symbols directly indicate some ICD codes.

3 https://pypi.org/project/jieba/.

4 Available at: https://pinyin.sogou.com/dict/cate/index/132.

5 https://clefehealth.imag.fr/?page_id=185

6 For CodiEsp dataset, we did not let $f_s$ take 120 and 160, as they resulted in the same subsets of data as those resulted from 100 and 140 respectively.

7 Theoretically, a higher *AUC* do not necessarily lead to a higher *F*1. When $f_s$ equaled 20, we tuned the calibration threshold, and found that the *BERT* variants did not result in *Micro-F*1 higher than that resulted by *BoW*. Details are given in Additional file 2.

8 Merely key unigram features were used.
